# Impaired antigen-specific B-cell responses after Influenza vaccination in kidney transplant recipients receiving co-stimulation blockade with Belatacept

**DOI:** 10.3389/fimmu.2022.918887

**Published:** 2022-07-28

**Authors:** Vincent Pernin, Maria Meneghini, Alba Torija, Thomas Jouve, Arnaud Del Bello, Iván Sanz-Muñoz, Jose Maria Eiros, Laura Donadeu, Carol Polo, Francisco Morandeira, Sergio Navarro, Cristina Masuet, Alexandre Favà, Moglie LeQuintrec, Nassim Kamar, Elena Crespo, Oriol Bestard

**Affiliations:** ^1^ Kidney Transplant Unit, Nephrology Department. Montpellier University Hospital, Montpellier, France; ^2^ Kidney transplant Unit. Nephrology Department. Vall d’Hebron Hospital Universitari, Vall d’Hebron Institut de Recerca (VHIR), Universitat Autònoma de Barcelona, Barcelona, Spain; ^3^ Laboratoryof Nephrology and Transplantation. Vall d’Hebron Institut de Recerca (VHIR), Universitat Autònoma de Barcelona, Barcelona, Spain; ^4^ Kidney transplant Unit, Nephrology Department. Grenoble University Hospital, Grenoble, France; ^5^ Centro Nacional de Gripe de Valladolid, Universidad de Valladolid, Valladolid, Spain; ^6^ Centro Nacional de Gripe, Valladolid Universidad de Valladolid, Valladolid, Spain; ^7^ Department of Microbiology and Parasitology, Rio Hortega University Hospital, University of Valladolid, Valladolid, Spain; ^8^ Kidney Transplant Unit, Nephrology Department, Bellvitge University Hospital, Barcelona, Spain; ^9^ Immunology Department, Bellvitge University Hospital, Barcelona, Spain; ^10^ Department of Preventive Medicine, Bellvitge University Hospital, Barcelona, Spain

**Keywords:** kidney transplantation, co-stimulation blockade, memory B cells, influenza vaccination, calcineurin inhibitors, T follicular helper (Tfh) cell

## Abstract

Emerging data suggest that costimulation blockade with belatacept effectively controls humoral alloimmune responses. However, whether this effect may be deleterious for protective anti-infectious immunity remains poorly understood. We performed a mechanistic exploratory study in 23 kidney transplant recipients receiving either the calcineurin-inhibitor tacrolimus (Tac, n=14) or belatacept (n=9) evaluating different cellular immune responses after influenza vaccination such as activated T follicular Helper (Tfh), plasmablasts and H1N1 hemagglutinin (HA)-specific memory B cells (HA^+^mBC) by flow-cytometry, and anti-influenza antibodies by hemagglutination inhibition test (HI), at baseline and days 10, 30 and 90 post-vaccination. The proportion of CD4+CD54RA-CXCR5+ Tfh was lower in belatacept than Tac patients at baseline (1.86%[1.25-3.03] *vs* 4.88%[2.40-8.27], p=0.01) and remained stable post-vaccination. At M3, HA^+^mBc were significantly higher in Tac-treated patients (0.56%[0.32-1.49] *vs* 0.27%[0.13-0.44], p=0.04) and correlated with activated Tfh numbers. When stratifying patients according to baseline HA^+^mBc frequencies, belatacept patients with low HA^+^mBC displayed significantly lower HA^+^mBc increases after vaccination than Tac patients (1.28[0.94-2.4] *vs* 2.54[1.73-5.70], p=0.04). Also, belatacept patients displayed significantly lower seroprotection rates against H1N1 at baseline than Tac-treated patients (44.4% *vs* 84.6%) as well as lower seroconversion rates at days 10, 30 and 90 after vaccination (50% *vs* 0%, 63.6% *vs* 0%, and 63.6% *vs* 0%, respectively). We show the efficacy of belatacept inhibiting T-dependent antigen-specific humoral immune responses, active immunization should be highly encouraged before starting belatacept therapy.

## Introduction

The calcineurin inhibitors (CNI), specifically tacrolimus, are the main immunosuppressant agents used in organ transplantation worldwide (1). CNIs abrogates the production of IL2 cytokine that drives T-cell activation and proliferation by inhibiting the activation of calcineurin induced by the engagement of the T-cell receptor with the peptide-MHC complex. More recently, the co-stimulation blockade immunosuppressant Belatacept (cytotoxic T lymphocyte-associated antigen 4 (CTLA4)-Ig; Bristol Myers Squibb), a human fusion protein which binds to surface costimulatory ligands (CD80 and C86) on antigen presenting cells (APCs) and thus, inhibits their interaction with CD28, consequently abrogating T-cell activation has been developed and can be used as an alternative of CNI in kidney transplantation (2).

The use of belatacept in human kidney transplantation as maintenance immunosuppression has shown to effectively prevent allograft rejection while improving renal function, together with a better cardio-vascular and metabolic profile as compared to a CNI based regimen, ultimately providing superior long-term graft survival (3, 4). Notably, while higher T-cell mediated rejection (TCMR) rates have been described in belatacept-treated patients (5), a remarkable ability to inhibit humoral alloimmunity by means of reduced *de novo* donor-specific anti-HLA antibody (DSA) formation and antibody-mediated rejection has also been described (6, 7). This effect is suggested to be driven by different mechanisms such as abrogation of antigen-specific T follicular Helper (Tfh) interaction with B cells, prevention of germinal center activation and a direct inhibition of donor-specific antibody-producing plasma cells (6, 8, 9). Importantly, while modulation of these humoral alloimmune responses is beneficial to preserve graft survival, data are lacking on how protective humoral immune responses against infectious antigens, such as viral antigen provided by vaccination, is influenced by belatacept.

Seasonal Influenza infection is a major public health problem causing significant morbidity and mortality particularly among more vulnerable patients such immunosuppressed solid organ transplants (SOT) (10) (11), in whom suboptimal humoral immune response seems to occur due to chronic immunosuppression (12–14).. Notably, an early expansion of Tfh cells, and especially of activated Tfh (*CD4^+^CD45RA^-^CXCR5^+^ICOS^+^PD1^+^)* has been shown to influence a better vaccination response (15, 16). Therefore, whether patients receiving belatacept may display a particularly impaired immune response after active Flu immunization has not been investigated yet.

In this study, we investigated the dynamics of Tfh and antigen-specific antibody responses by hemagglutination inhibition test and B-cell immune responses in belatacept-treated kidney transplant patients compared to standard of care Tacrolimus (Tac)-treated patients, after receiving a T-cell dependent booster vaccination against Influenza virus.

## Methods

### Study population and study design

Twenty-three consecutives adult kidney transplant recipients were recruited at the kidney transplant units at Bellvitge University Hospital (Barcelona, Spain), and Toulouse University Hospital (Toulouse, France) in 2017-2018 fall periods. Patients were included if they were scheduled to receive the influenza vaccine, had a functioning kidney allograft for more than 1 year and were receiving maintenance immunosuppression regimen based on either the CNI tacrolimus (Tac Group) or Belatacept (BELA Group) (supplemental material).

Main exclusion criteria were documented egg allergy, acute febrile illness during the 7 days preceding vaccination, previous allergic reaction to influenza vaccine and acute rejection in the last 6 months.

Serum and peripheral blood mononuclear cell (PBMC) samples were collected prior to (Day 0) and at 10 days, 1 month and 3 months after vaccination for immune analyses. The local institutional review board approved the study, and all participants gave written informed consent.

### Influenza vaccines

All patients received a single 0.5mL intramuscular injection of the trivalent vaccine (CHIROMAS®, Seqirus, UK or VAXIGRIP®, Sanofi Pasteur, France) in the deltoid muscle. These vaccines contain 15ug of hemagglutinin (HA) antigen from the following virus strains: A/Michigan/45/2015(H1N1)pdm09-like, A/HongKong/4801/2014(H3N2) and B/Brisbane/60/2008.

### Assessment of influenza (H1N1-HA)-specific memory B cells

A recombinant H1N1-HA from the virus strain A/Michigan/45/2015(H1N1)pdm09-like (eENZYME, USA) was chemically labeled with Alexa Fluor 647 (Alexa Fluor™ NHS ester, Molecular Probes, Invitrogen) following the manufacturer’s instructions, for the detection of HA-specific Memory B cells by flow-cytometry analysis as described below. No biotinylation is necessary, as direct conjugation with the protein is achieved as reported on manufacturer’s instructions. The degree of labeling was determined by spectrophotometry (Supplemental material). One µg of dye was used to label 10 µg of HA and stored at a concentration of 30 µg/ml in PBS.

As a negative control, Human Serum Albumin (Equipos Medico-biológicos, Barcelona, Spain) labelled with Alexa Fluor 647 with the same kit (Alexa Fluor™ NHS ester, Molecular Probes, Invitrogen) was used to control the absence of unspecific B cell interaction with the dye (**Supplementary Fig 1B**) in all patients at baseline. We did not detect specific binding to albumin in any case.

### Flow cytometry analyses

Peripheral blood mononuclear cells (PBMC) were isolated by density gradient ficoll separation and conserved in liquid nitrogen until use. PBMC were thawed, pretreated with saturating concentration of human aggregated immunoglobulins to block FcγR and then stained with the following markers: CD19 PerCPVio700 (Miltenyi Biotec), CD27 PEVio770 (Miltenyi Biotec), IgD FITC (BD Biosciences), CD38 APCR-700 (BD Biosciences), CD45RA FITC (BD Biosciences), CD4 PEVio770 (BD Biosciences), CD185-CXCR5 PE (Biolegend), PD1 A-647 (Biolegend), CD278- ICOS PerCPVio700 (Miltenyi Biotec) and H1N1-HA labeled with Alexa Fluor 647 as previously described in order to define the T follicular helper and the B lymphocytes subsets. The Live/Dead viability kit (Thermofischer, Invitrogen) was used to discriminate dead cells.

Importantly, 10ul per million of PBMC of Alexa Fluor 647 labeled HA or human albumin was added after Live/Dead viability kit and Fc Blocker Biolegend and stored at 4° in the dark for 15 minutes before staining with the rest of antibodies according to manufacturer’s instructions. One million of PBMC was the minimum of cells used in each experiment.

After staining, cells were acquired using the Gallios flow cytometer (Beckman Coulter). All proportions of each lymphocyte subset are expressed as a percentage of the total lymphocyte count.

The gating strategy for Tfh and BC subsets are shown is the **Supplementary Figures 1**, **2** respectively together with representative examples for both BELA and TAC patient samples.

### Hemagglutination inhibition assay

The analysis of the presence of haemaglutinant antibodies against influenza H1N1 lineage was performed by haemaglutination inhibition assay (HI), performed using the same strain contained in the 2017-2018 vaccine, as previously described (17), according to WHO guidelines (*WHO Global Influenza, Surveillance Network Manual for the laboratory diagnosis and virological surveillance of influenza. 2011*). The test was performed in 96-microV well plates, serum samples were pre-treated with RDE (Receptor Destroying Enzyme; Denka Seiken, Tokyo, Japan) and then adsorbed with hen red blood cells for eliminating the unspecific inhibitors from blood. Results interpretation was performed using classical European Medicament Agency (*EMA)* criteria for the evaluation of vaccine efficacy (*EMA; Note for guidance on harmonization of requirements for influenza vaccines (CPMP/BWP/214/96)* (1997)): seroconversion was defined as either post vaccination serum titer ≥1/40 with pre-vaccination negative HI test or significant increase of antibody titer of at least 1 fourfold increase after vaccination. Seroprotection was defined as an antibody titer ≥1/40 (18, 19). Geometric Mean Titers (GMT) were also analyzed in different groups of patients and timepoints.

### Statistics

We performed two-groups comparisons between the CNI and BELA groups using the Wilcoxon test for continuous covariates and the Fisher exact test for categorial covariates. For the temporal evolution, we considered the baseline differences in lymphocytes’ phenotypes using an ANCOVA model predicting the final M3 lymphocytes subpopulations, adjusting for the baseline subpopulations and for the treatment. P-values <0.05 were considered statistically significant. Analyses were performed using SPSS version 26 software and the R software version 1.0.12 for the temporal evolution analysis. Graphs were generated using GraphPad Prism version 8.0 software (Graphpad Software, San Diego, California).

## Results

### Baseline characteristics of the study population

Twenty-three kidney transplant recipients were included in this study, 9 patients were receiving Belatacept (Bela Group) and 14 a tacrolimus-based immunosuppressive regimen (Tac group). Patients were immunized with the trivalent influenza vaccine (CHIROMAS® n= 20 or VAXIGRIP® n=3). As described in **Table 1**, there was no major difference regarding main clinical, demographic or immunological characteristics between all patients of the study, but Bela treated patients had a longer time since transplantation. There was no acute rejection event nor *de novo* DSA formation in any patient after vaccination during the study follow-up. Also, none of the patients developed a Flu infection during the study follow-up.

**Table 1:** Main characteristics of the study population.

	Belatacept (n=9)	Tacrolimus (n=14)	p value
Time after transplantation (years)	9.67 ± 4.19	3.5 ± 4.27	0.003
Recipient age (years)	60.8 ± 10.3	54.9 ± 12.3	0.25
Recipient gender (Male) (n, %)	8 (89%)	9 (64%)	0.34
Donor age (years)	60.2 ± 17.5	54.8 ± 13.4	0.51
**Transplant type** Living (n, %) DBD/DCD (n, %)	1 (11%) 6 (67%)/2 (22%)	1 (7%) 11 (79%)/2 (14%)	0.82
Transplant number (1^st^) (n, %)	7 (78%)	10 (83%)	0.48
Time on dialysis (months)	17.8 ± 18.3	34.3 ± 25	0.18
**Cause of ESRD** (n, %) Glomerular diseases Diabetic nephropathy Polycystic kidney disease Vascular/Hypertension Others Unknown	4 (44%) 0 1 (11%) 1 (11%) 3 (33%) 0	5 (36%) 1 (7%) 3 (21%) 2 (14%) 1 (7%) 2 (14%)	0.47
**Relevant comorbidities** (n, %) Diabetes Hypertension Heart failure Previous splenectomy	0 6 (67%) 1 (11%) 0	2 (15%) 11 (85%) 2 (15%) 0	0.49 0.61 1
**Induction treatment** (n, %) None Basiliximab rATG	1 (14%) 7 (78%) 1 (14%)	0 11 (79%) 3 (21%)	0.39
MMF dose (mg/d)	826 ± 332	881 ± 227	0.66
Prior anti-flu vaccination (n, %)	7 (78%)	12 (86%)	1
Type of vaccination (CHIROMAS)	3 (33%)	0	0.047
cPRA at time of vaccination (%)	10.2 ± 31	10.9 ± 22	0.95
DSA at time of vaccination (n, %)	0	2 (14%)	0.17
*De novo* DSA after vaccination (n, %)	0	0	1
Acute rejection prior to vaccination (n, %)	1 (12.5%)	2 (13%)	1
Acute rejection after vaccination (n, %)	0	0	1
eGFR at time of vaccination (mL/min/1.73m2)	54.9 ± 22	63.9 ± 15	0.34

M, male; DBD, donation after brain death; DCD, donation after cardiac death; rabbit antithymoglobulin; MMF, Mycophenolate mofetil DGF, delayed graft function; DSA, donor-specific antibodies; eGFR, estimated glomerular filtration rate; ESRD, end-stage renal disease; cPRA, calculated panel reactive antibodies.

### Kinetics of circulating Memory and follicular Helper T cells after influenza vaccination

**Figure 1:**
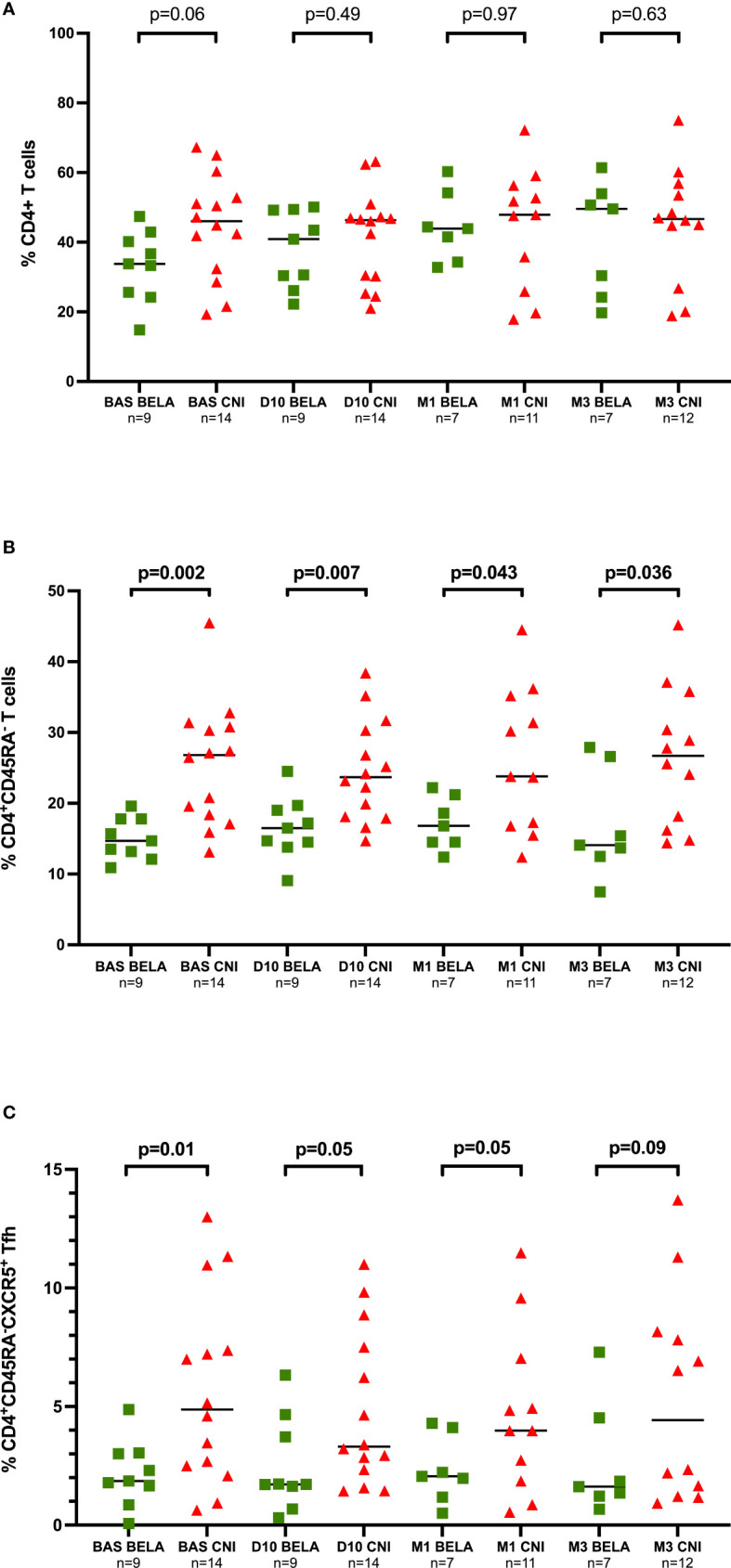
Kinetics of different T-cell subsets at different time points prior and after Influenza immunization. **Figure 1A**. Proportion of CD4^+^ T cells. **Figure 1B**. Proportion of CD4^+^CD45RA^-^ memory T cells, **Figure 1C**. Proportion of CD4^+^CD45RA^-^CXCR5^+^ Tfh cells. *The proportions of CD4^+^ T cells, CD4^+^CD45RA^-^ memory T cells and CD4^+^CD54RA^-^CXCR5^+^ Tfh cells are expressed as a percentage in total lymphocytes. Comparison of proportions between the two groups at each time point were analyzed using a Mann-Whitney Test*.

Total lymphocyte counts were not different in both groups both before and after immunization (data not shown). As shown in **Figure 1**, at baseline, the proportion of CD4+ T cells, CD4+CD45RA- memory T cells and CD4+CD45RA-CXCR5+ Tfh cells were lower in the Bela group as compared to the Tac group (33.8%[24.9-41.6] *vs* 46.1%[31.5-54.7] CD4+, p=0.06; 14.7%[12.7-17.8] *vs* 26.8%[18.1-30.9] CD4+CD45RA-, p=0.002 and 1.86%[1.25-3.03] *vs* 4.88%[2.40-8.27] CD4+CD45RA-CXCR5+, p=0.01, for Bela and Tac groups respectively). After immunization, the proportion of CD4+ T cells, CD4+CD45RA- memory T cells and CD4+CD45RA-CXCR5+ Tfh did not significantly change over time between groups as compared to baseline.

**Figure 2:**
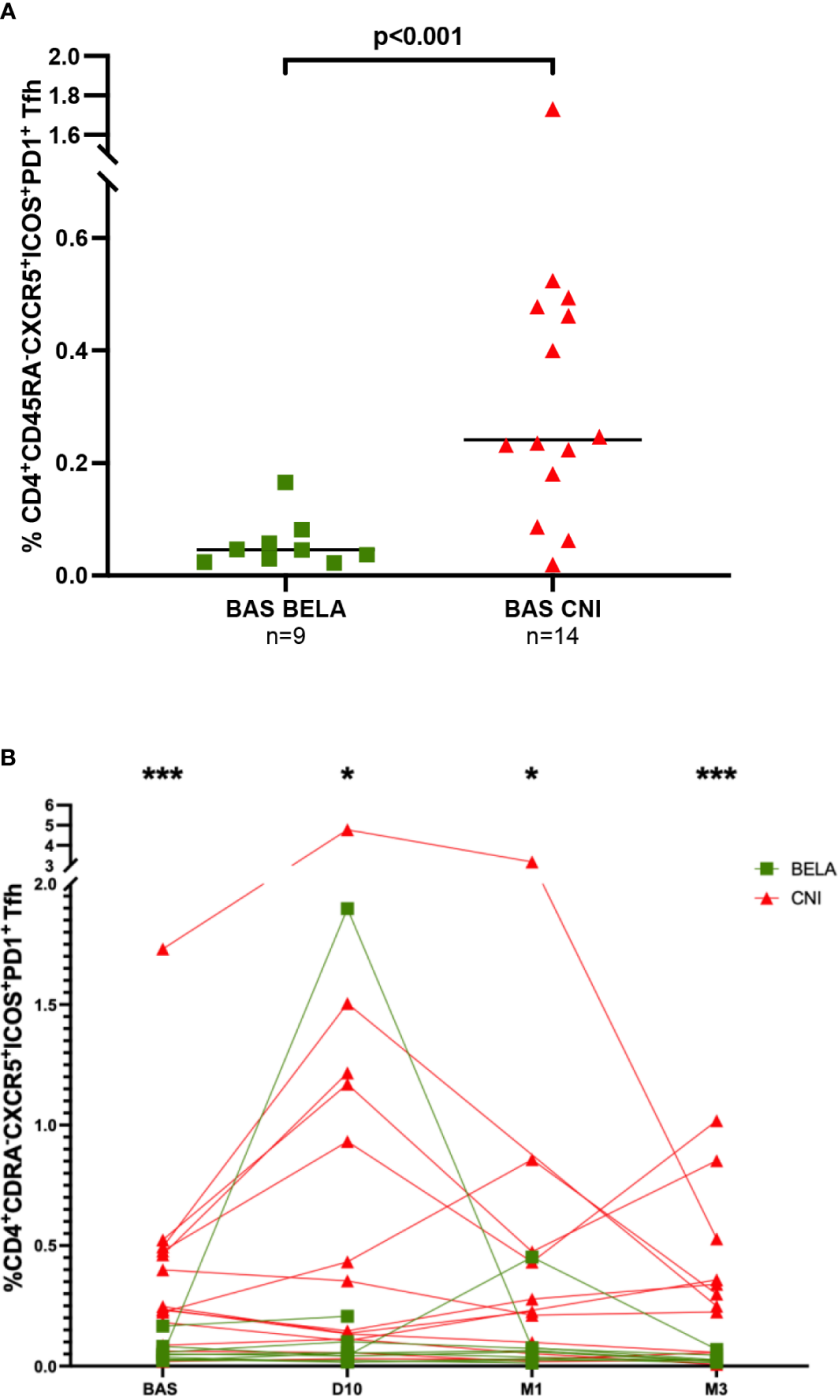
Comparison of the percentage of CD4^+^CD45RA^-^CXCR5^-^ICOS^+^PD1^+^ activated Tfh between the two study groups. **Figure 2A**. Baseline percentages of CD4^+^CD45RA^-^CXCR5^-^ICOS^+^PD1^+^activated Tfh in the two study groups. **Figure 2B**. Kinetics of CD4^+^CD45RA^-^CXCR5^-^ICOS^+^PD1^+^ activated Tfh after influenza immunization in the two study groups. The proportion of CD4^+^CD45RA^-^CXCR5^-^ICOS^+^PD1^+^ Tfh cells is expressed as a percentage in total lymphocytes in both figure 2A and 2B. Comparison of proportions between the two groups at each time point were analyzed using a Mann-Whitney Test. * p<0.05, ** p<0.01, ***p<0.001.

When we focused on activated CD4+CD45RA-CXCR5+ICOS+PD1+ Tfh, at baseline, significantly lower numbers were observed in the Bela group than in the Tac group (0.05%[0.03-0.07] *vs* 0.24%[0.16-0.48], p<0.001) (**Figure 2A**). Notably, while these numbers did not change at any time point after vaccination in Bela patients but in one patient, there was a significant increase of activated Tfh among 6/14 patients in the Tac group at day 10 (**Figure 2B**). Activated Tfh remained higher in the Tac group at D10 (0.05%[0.02-0.15] *vs* 0.25%[0.11-1.18], p=0.019 for Bela and Tac group respectively), M1 (0.04%[0.02-0.07] *vs* 0.23%[0.05-0.47] respectively, p= 0.027), and M3 (0.02%[0.02-0.03] *vs* 0.27%[0.06-0.49] respectively, p=0.004). The same feature was observed when the percentage of activated Tfh was assessed over total Tfh cells (data not shown).

### Kinetics of different B-cell subsets after influenza vaccination

#### Total CD19+ B cells and other B-cell subsets

As illustrated in supplemental **Figure 3**, the percentages of CD19+ B cells, CD19+CD27+ memory B cells (mBC), CD19+CD27+IgD- switched mBc and CD19+CD27+IgD+ unswitched mBC were similar between groups, both at baseline and at the different time points after vaccination.

#### Total CD19^+^CD27^+^CD38^hi^ plasmablasts

**Figure 3:**
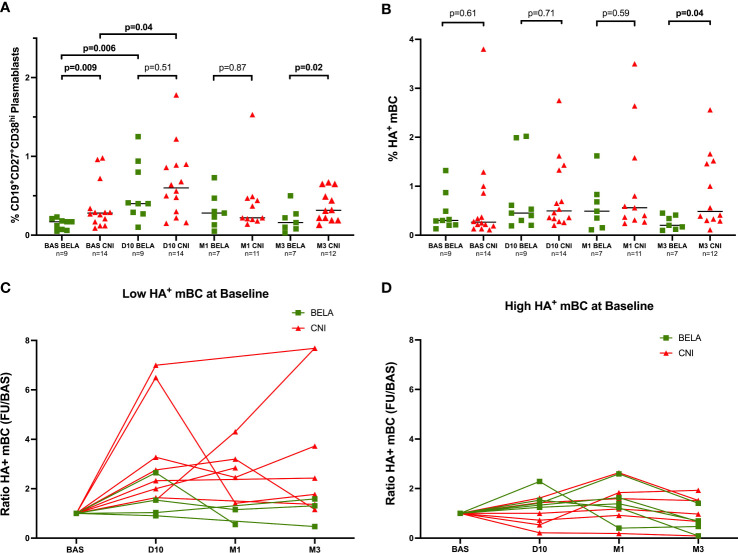
Kinetics of circulating Plasmablasts and Influenza-specific B-cell subsets prior and after Influenza immunization. **Figure 3A** Proportion of CD19^+^CD27^+^CD38^hi^ plasmablasts. **Figure 3B**. Proportion of CD19^+^CD27^+^HA^+^ mBc. **Figure 3C**. Analysis of HA^+^mBC expansion after immunization in patients with High preformed HA+mBc at baseline. **Figure 3D**. Analysis of HA^+^mBC expansion after immunization in patients with Low preformed HA+mBc at baseline. *The proportions of CD19^+^CD27^+^CD38^hi^ cells **(Figure 2A)** and CD19^+^CD27^+^H1N1^+^ cells **(Figure 2B)** are expressed as a percentage in total lymphocytes in both **Figures 3A, 3B**. The ratio of H1N1^+^CD19^+^CD27^+^ mBC at D10, M1 and M3 after immunization **(Figures 3C, 3D)** = % H1N1^+^CD19^+^CD27^+^ mBC during follow-up/% H1N1^+^CD19^+^CD27^+^ mBC at baseline. Comparison of proportions between the two groups at each time point were analyzed using a Mann-Whitney Test*.

At baseline, the percentage of CD19^+^CD27^+^CD38^hi^ plasmablasts was significantly lower in the Bela group than in the Tac group (0.17%[0.07-0.20] *vs* 0.28%[0.16-0.44], p=0.009) (**Figure 3A**). At day 10 after vaccination, a significant increase of plasmablasts in both groups was observed (0.40%[0.28-0.87] *vs* 0.17%[0.07-0.20], p=0.006 and 0.60%[0.28-0.89] *vs* 0.28%[0.16-0.44], p=0.04, at D10 *vs* Baseline in the Bela and Tac groups, respectively), and the proportion of plasmablasts became similar between the two groups at D10 and at M1. While after day 10 the proportion of plasmablasts progressively decreased returning to baseline levels, at M3 Bela patients showed significantly lower levels than the Tac group (0.13%[0.04-0.26] *vs* 0.31%[0.21-0.57], p=0.02).

A moderate but significant correlation between the variation of CD4^+^CD45RA^-^CXCR5^+^ICOS^+^PD1^+^ activated Tfh between Baseline to D10 (ΔActivated Tfh= %Activated Tfh at D10 - %Activated Tfh at baseline) and the percentage of plasmablasts at D10: Pearson r=0.43, p=0.04 in all patients (**Supplemental Figure 4**).

#### H1N1 HA-specific CD19^+^CD27^+^ memory B cells

The levels of circulating HA-specific mBc (HA^+^CD19^+^CD27^+)^ at baseline ranged between 0.11% to 3.8%, and were similar between both groups (0.25% of CD19+ cells [0.19-0.86] *vs* 0.30%[0.20-0.68], p=0.61, in the Tac and Bela groups, respectively). Notably, while these numbers did not differ between groups at D10 and M1 (0.50%[0.33-1.36] *vs* 0.45%[0.25-1.3] at D10, p=0.71 and 0.56%[0.31-1.58] *vs* 0.49%[0.15-0.83] at M1, p=0.59 in Tac and Bela, respectively), a significantly higher increase of HA^+^CD19^+^CD27^+^ mBc in the Tac group compared to Bela patients was observed at M3 (0.56%[0.32-1.49] *vs* 0.27%[0.13-0.44] respectively, p=0.04) (**Figure 3B**).

To further investigate the effect of vaccination on HA^+^CD19^+^CD27^+^ mBC, we stratified patients into two subgroups: High HA^+^CD19^+^CD27^+^ mBC or Low HA^+^CD19^+^CD27^+^ mBC according to the median percentage of HA^+^CD19^+^CD27^+^ mBc at baseline (HA^+^CD19^+^CD27^+^ mBC=0.29%), suggesting the presence or absence of a preformed mBc response already at baseline. The expansion of HA^+^CD19^+^CD27^+^mBC after vaccination was analyzed as the ratio of HA^+^CD19^+^CD27^+^ mBC after immunization over the numbers of HA^+^CD19^+^CD27^+^mBC at baseline. As shown in **Figure 3C**, among the Low HA^+^CD19^+^CD27^+^mBC group, a significant higher increase of HA^+^CD19^+^CD27^+^mBC was observed in the Tac group as compared to the Bela group (Ratio HA^+^CD19^+^CD27^+^mBC: 2.54[1.73-5.70] *vs* 1.28[0.94-2.4] at D10, p=0.05; 2.85[1.93-3.75] *vs* 0.85[0.55-1.15] at M1, p=0.09 and 2.43[1.36-7.68] *vs* 0.92[0.49-1.52] at M3, p=0.04 in the Tac and Bela groups, respectively). Conversely, no significant differences were observed between groups within patients showing High HA^+^CD19^+^CD27^+^ mBC (**Figure 3D**).

Adjusting for the continuous cell subsets frequencies at baseline, HA^+^CD19^+^CD27^+^ mBc at M3 was the only cell population associated with a treatment effect (baseline effect +0.46% p<0.001; treatment effect [Bela VS Tac]: -0.49% [p=0.028], p=ns for all other populations).

### Serological responses after influenza vaccination

**Figure 4:**
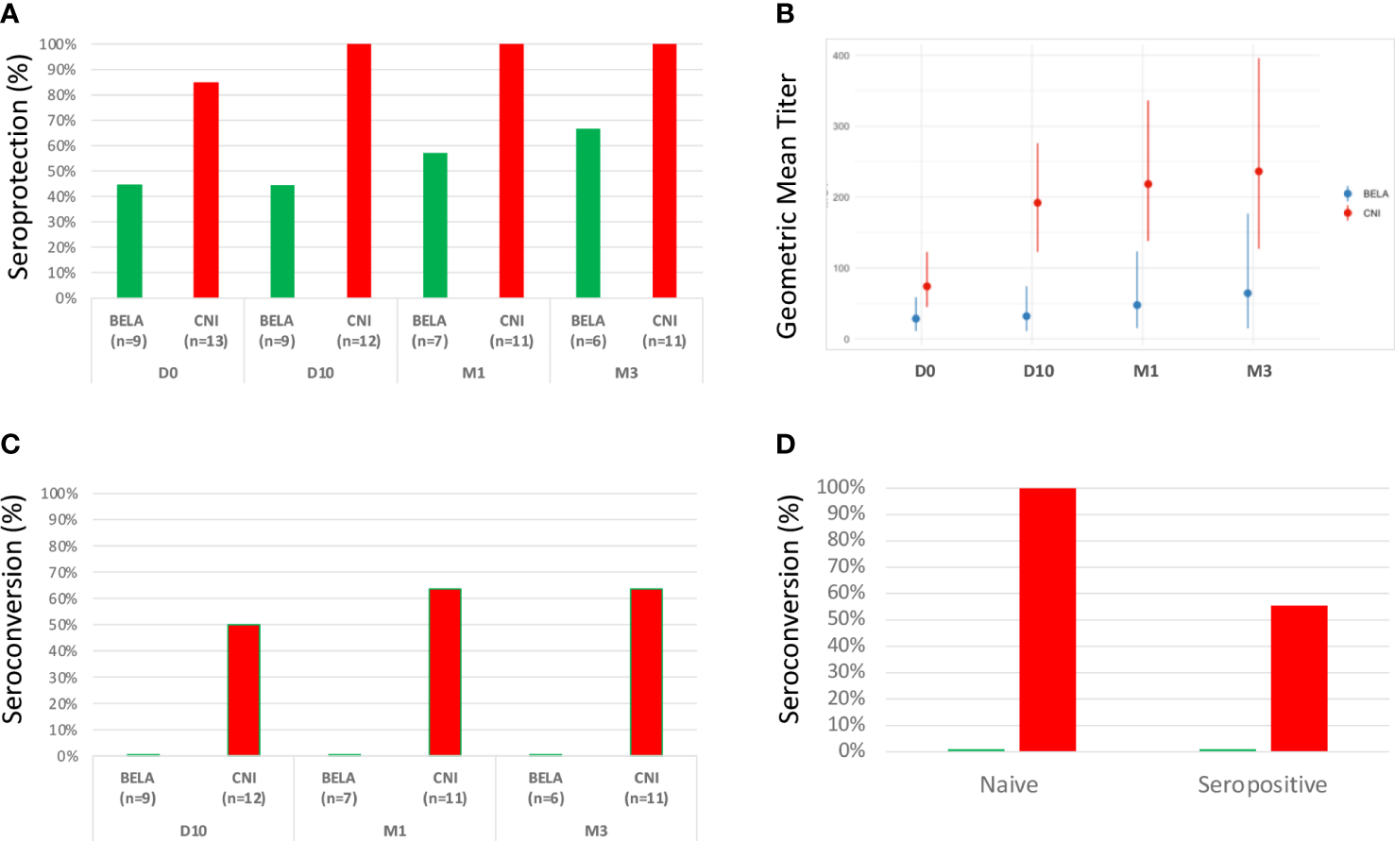
Serological responses after Influenza vaccination. **Figure 4A**. Seroprotection rate at baseline and evolution after vaccination. **Figure 4B**. Kinetics of H1N1 antibody titers (GMTs) before and after vaccination. **Figure 4C**. Seroconversion rate after vaccination. **Figure 4D**. Comparison of seroconversion rates at M3 between naïve and seropositive patients prior to vaccination according to each type of immunosuppressant.

At baseline, despite similar previous vaccination rates in both groups, Influenza seroprotection rate was higher in the Tac group (84,6%) as compared to the Belatacept group (44,4%) (**Figure 4A**). After vaccination, H1N1 IgG seroprotection rate reached 100% already at D10 and was maintained until M3 in the Tac group, whereas only 66.7% of seroprevalence rate was observed in Bela patients at M3. Accordingly, the geometric median titer (GMT) of H1N1 IgG antibodies at baseline was also significantly higher in the Tac group (74.0[44.9-122.6] *vs* 28.3[10.8-58.8] in the BELA group, p=0.04, **Figure 4B**). Indeed, the Tac group displayed a higher increase of GMT at the distinct time points after vaccination as compared to BELA patients: 191.8[122.6-275.8] *vs* 31.8[10.8-74.1] p=0.003, 218.2[137.9-336.2] *vs* 47.6 [15.2-123.4], p=0.008 and 236.2[127-396.1] *vs* 64.3[14.9-176.6], p=0.05, at D10, M1 and M3 in Tac and Bela, respectively) (**Figure 4B**). While seroconversion occurred in 50%, 63.6% and 63.6% of patients at D10, M1 and M3 respectively in the Tac group, none of the Bela patients showed seroconversion in the BELA group during follow-up (**Figure 4C**). Notably, and similarly to what was observed when stratifying patients with low/High preformed HA^+^CD19^+^CD27^+^ mBc, within serologically naïve patients (H1N1 IgG <1/40) at baseline, seroconversion at M3 occurred in 2/2 (100%) patients of the CNI group and in none (0/3 [0%]) in the group Bela group. Likewise, among patients with detectable H1N1 IgG titers (≥1/40) at baseline, seroconversion at M3 was achieved in 5/9 (55.6%) Tac patients but in none of the BELA group (0/3[0%]) (**Figure 4D**).

## Discussion

In this study, we show that the generation of a protective humoral immune memory response in kidney transplant patients receiving a T-cell dependent booster vaccination such as that against Influenza virus is significantly impaired among patients receiving co-stimulation blockade with belatacept as compared to patients on a CNI-based regimen with tacrolimus. Indeed, significantly lower seroprotection rates and memory B-cell (mBc) frequencies specific to influenza virus after vaccination were observed in belatacept-treated patients as compared to patients on tacrolimus.

While the inhibitory effect of belatacept on the humoral alloimmune memory response among SOT patients has been shown at the clinical, experimental, and *in vitro* levels its effect on other humoral immune responses (such as vaccination-induced immunization) among SOT patients has not been reported. This inhibitory effect on allo-immunity seems multifactorial, e.g., preventing the cross-talk between activated Tfh cells and B cells (6–8), ultimately preventing germinal center activation and DSA formation. Indeed, in some autoimmune diseases, previous studies have shown an altered humoral immune response after influenza vaccination. In 2013, Ribeiro et al. (20) reported that seroprotection was significantly reduced after the pandemic 2009 influenza A/H1N1 vaccine in a cohort of rheumatoid arthritis (RA) patients treated with the costimulation blocker abatacept as compared to age-matched RA-patients treated with methotrexate (9% *vs* 58%, p=0.0006). Kapetanovic et al. (21) showed a seroconversion rate in only 20% of RA patients treated with abatacept as compared with 42% of patients on methotrexate and 53% on anti-TNF monotherapy after H1N1 influenza vaccine. Similarly, during recent vaccination campaign against SARS-COV2, kidney transplant patients receiving belatacept maintenance therapy developed significantly inferior serological response after vaccination even if no clear effect was observed on risk of severe disease after natural infection (22, 23).

Tacrolimus also reduces significantly the number of circulating Tfh and their activation capacity (24). In this study, we show that belatacept-treated patients display significantly lower numbers of circulating Tfh cells than patients on CNI-based immunosuppression and failed to undergo a significant expansion of these activated TFH cells after vaccination, as compared to tacrolimus-treated patients. While the global percentages of mBc with distinct phenotypes (such as switched and unswitched mBc) and antigen specificities were similar between treatment groups at baseline, belatacept-treated patients showed a significantly lower percentage of plasmablasts at baseline than CNI patients. As previously reported after influenza vaccination in healthy individuals (15, 16), we also observed a positive correlation between the early expansion of activated Tfh cells and the increase of plasmablasts at D10.

Interestingly, patients of the study had already received a previous vaccination against the Influenza virus or had been naturally immunized through a viral infection in the past, therefore most patients were seroprotected already at baseline. However, while the level of HA^+^mBC at baseline was variable and ranged between 0.11% and 3.8% in our patients, their expansion at 3 months after vaccination was significantly lower in the BELA than in the CNI group. Furthermore, belatacept seemed to particularly inhibit the generation of new HA^+^mBC as though patients with low HA+mBc levels at baseline showed significantly lower expansion of these cells at M3 than CNI patients with the same low pre-vaccine levels. Conversely, in patients with high preformed HA^+^mBC levels at baseline, the level of HA^+^mBC did not significantly change after vaccination in both groups. These results are in line with experimental models of alloimmune sensitization in which belatacept seems to be especially capable to reverse a T-dependent humoral alloimmune response when the treatment is given during the initial period after alloimmunization, whereas once a complete germinal center activation has been generated, belatacept has an impact similar to that of CNIs (25, 26).

When assessing serological response to vaccination by means of specific HI test, we observed that even with a similar history of previous flu vaccinations/infections, patients receiving belatacept displayed inferior seroprotection at baseline as well as lower percentage of seroconversion and antibody titers after vaccination. We then stratified patients according to baseline seroprotection, we detected a similarly poor response to vaccination both in patients with negative (<1/40) or positive (≥1/40) baseline antibody titer.

We acknowledge some limitations: the small sample size, the focus on the H1N1 strain (and not other hemagglutinin antigens contained in the vaccine) as though previous studies have reported that such humoral responses may differ according to the type of hemagglutinin (13, 27). It must be noted that two different vaccines were used in the present study, however both had the same HA and viral strains composition and no differences were observed in B cell nor antibody response according to the type of vaccine. Also, for Tfh analysis we analyzed the kinetics of total circulating Tfh and their activation markers but we did not measure an antigen-specific response. However, the consistent differences observed between the two groups evaluated in different cell compartments reinforce the biological plausibility of our data. Furthermore, all patients of the study were also receiving MMF as concomitant immunosuppressant and previous reports have suggested a deleterious effect of MMF on the generation of humoral immune responses after active immunization (12, 14, 28). Thus, although both study groups were receiving the same MMF doses, we cannot exclude a synergistic effect of MMF and belatacept in impairing the humoral response, as compared to patients receiving a combination of tacrolimus and MMF. The main clinical difference between study group was time since transplantation, being longer in BELA patients. Even if we cannot exclude that it might be a confounder for our results, it has been shown that shorter time after transplantation is a risk factor for lower response to vaccination, we do not think this factor explains the differences observed between the two groups of treatment since the increased immunosuppressive load is commonly observed during the first 6 months after transplantation and not in such later time points as our patient population was investigated (29).

In conclusion, we showed that, unlike CNI-treated kidney transplant patients, patients on belatacept display significantly lower circulating Tfh and plasma cell levels and have a significantly impaired humoral immune memory response after influenza vaccination, especially those with low preformed antigen-specific mBC. While further investigation is warranted, our data suggest that patients considered to be treated by co-stimulation blockade with belatacept are highly encouraged to receive active immunization prior to this therapy in order to increase their baseline anti-viral immune memory protection and ultimately minimize the risk of subsequent opportunistic infections.

## Data availability statement

The original contributions presented in the study are included in the article/**supplementary material**. Further inquiries can be directed to the corresponding author.

## Ethics Statement

The studies involving human participants were reviewed and approved by ethic committee of Bellvitge University Hospital. The patients/participants provided their written informed consent to participate in this study.

## Author contributions

EC and OB had designed the study. VP, MM, IS-M, TJ, AT, JME, LD performed the experiments. EC, OB, MM, CP, FM, SN, CM, AF, AB and NK had enrolled patients in the study. OB, EC, VP, MM, IS-M, TJ, had participated to data analysis and wrote the manuscript. All authors contributed to the article and approved the submitted version.

## Funding

This work was supported by the Instituto de Salud Carlos III (ISCIII) (grant numbers ICI14/00242 and PI16/01321, PI19/01710) and by the European Union’s Horizon 2020 Research and innovation program (grant agreement 754995). Also, this work was partly supported by the SLT002/16/00183 grant, from the Department of Health of the Generalitat de Catalunya by the call “Accioí instrumental de programes derecerca orientats en l’àmbit de la recerca i la innovacioí en salut.” The authors thank the Research Centers of Catalonia (CERCA) Programme/Generalitat de Catalunya for institutional support. OB was awarded with an intensification grant from the “Instituto de Salud Carlos III” [INT19/00051].

TJ received a fellowship grant from ESOT (European Society for Organ Transplantation).

## Acknowledgments

We acknowledge the assistance of our lab technicians and the biobank of our center for carefully management of all biological samples, and the staff of the flow cytometer technical support.

## Conflict of Interest

The authors declare that the research was conducted in the absence of any commercial or financial relationships that could be construed as a potential conflict of interest.

## Publisher’s note

All claims expressed in this article are solely those of the authors and do not necessarily represent those of their affiliated organizations, or those of the publisher, the editors and the reviewers. Any product that may be evaluated in this article, or claim that may be made by its manufacturer, is not guaranteed or endorsed by the publisher.
