# Corrigendum: Impaired antigen-specific B-cell responses after Influenza vaccination in kidney transplant recipients receiving co-stimulation blockade with Belatacept

**DOI:** 10.3389/fimmu.2024.1447503

**Published:** 2024-07-01

**Authors:** Vincent Pernin, Maria Meneghini, Alba Torija, Thomas Jouve, Arnaud Del Bello, Iván Sanz-Muñoz, Jose Maria Eiros, Laura Donadeu, Carol Polo, Francisco Morandeira, Sergio Navarro, Cristina Masuet, Alexandre Favà, Moglie LeQuintrec, Nassim Kamar, Elena Crespo, Oriol Bestard

**Affiliations:** ^1^ Kidney Transplant Unit, Nephrology Department. Montpellier University Hospital, Montpellier, France; ^2^ Kidney transplant Unit. Nephrology Department. Vall d’Hebron Hospital Universitari, Vall d’Hebron Institut de Recerca (VHIR), Universitat Autònoma de Barcelona, Barcelona, Spain; ^3^ Laboratoryof Nephrology and Transplantation. Vall d’Hebron Institut de Recerca (VHIR), Universitat Autònoma de Barcelona, Barcelona, Spain; ^4^ Kidney transplant Unit, Nephrology Department. Grenoble University Hospital, Grenoble, France; ^5^ Centro Nacional de Gripe de Valladolid, Universidad de Valladolid, Valladolid, Spain; ^6^ Centro Nacional de Gripe, Valladolid Universidad de Valladolid, Valladolid, Spain; ^7^ Department of Microbiology and Parasitology, Rio Hortega University Hospital, University of Valladolid, Valladolid, Spain; ^8^ Kidney Transplant Unit, Nephrology Department, Bellvitge University Hospital, Barcelona, Spain; ^9^ Immunology Department, Bellvitge University Hospital, Barcelona, Spain; ^10^ Department of Preventive Medicine, Bellvitge University Hospital, Barcelona, Spain

**Keywords:** kidney transplantation, co-stimulation blockade, memory B cells, influenza vaccination, calcineurin inhibitors, T follicular helper (Tfh) cell

In the published article, there was an error in **Table 1** as published. While it stated that 3 (33%) patients received the “Chiromas” vaccine, the correct information is that 3 (33%) patients in the belatacept group received the “Vaxigrip” vaccine. The corrected **Table 1** and its caption appear below.

**Table 1:** Main characteristics of the study population.

	Belatacept (n=9)	Tacrolimus (n=14)	p value
**Time after transplantation (years)**	**9.67 ± 4.19**	**3.5 ± 4.27**	**0.003**
Recipient age (years)	60.8 ± 10.3	54.9 ± 12.3	0.25
Recipient gender (Male) (n, %)	8 (89%)	9 (64%)	0.34
Donor age (years)	60.2 ± 17.5	54.8 ± 13.4	0.51
**Transplant type**			0.82
Living (n, %)	1 (11%)	1 (7%)	
DBD/DCD (n, %)	6 (67%)/2 (22%)	11 (79%)/2 (14%)	
Transplant number (1^st^) (n, %)	7 (78%)	10 (83%)	0.48
Time on dialysis (months)	17.8 ± 18.3	34.3 ± 25	0.18
**Cause of ESRD** (n, %)			0.47
Glomerular diseases	4 (44%)	5 (36%)	
Diabetic nephropathy	0	1 (7%)	
Polycystic kidney disease	1 (11%)	3 (21%)	
Vascular/Hypertension	1 (11%)	2 (14%)	
Others	3 (33%)	1 (7%)	
Unknown	0	2 (14%)	
**Relevant comorbidities** (n, %)			
Diabetes	0	2 (15%)	0.49
Hypertension	6 (67%)	11 (85%)	0.61
Heart failure	1 (11%)	2 (15%)	1
Previous splenectomy	0	0	
**Induction treatment** (n, %)			0.39
None	1 (14%)	0	
Basiliximab	7 (78%)	11 (79%)	
rATG	1 (14%)	3 (21%)	
MMF dose (mg/d)	826 ± 332	881 ± 227	0.66
Prior anti-flu vaccination (n, %)	7 (78%)	12 (86%)	1
Type of vaccination (Vaxigrip)	3 (33%)	0	0.047
cPRA at time of vaccination (%)	10.2 ± 31	10.9 ± 22	0.95
DSA at time of vaccination (n, %)	0	2 (14%)	0.17
*De novo* DSA after vaccination (n, %)	0	0	1
Acute rejection prior to vaccination (n, %)	1 (12.5%)	2 (13%)	1
Acute rejection after vaccination (n, %)	0	0	1
eGFR at time of vaccination (mL/min/1.73m2)	54.9 ± 22	63.9 ± 15	0.34

The authors apologize for this error and state that this does not change the scientific conclusions of the article in any way. The original article has been updated.

