# Supplementary material for: Impaired antigen-specific B-cell responses after Influenza vaccination in kidney transplant recipients receiving co-stimulation blockade with Belatacept
Source: Front Immunol. 2022 Jul 28;13:918887. doi: 10.3389/fimmu.2022.918887 (PMC9374104; doi:10.3389/fimmu.2022.918887)
Supplement: Supplementary file 1 [file DataSheet_1.docx]

Supplementary Material

**Immunosuppressive regimen of patients of the study**

For maintenance immunosuppression, all patients in the CNI-group received tacrolimus (once or twice daily formulations) with a stable trough level during the follow-up between 6-8ng/ml, associated with 500mg big of mofetil mycophenolate (MMF) and 5 mg/day of oral prednisone. In the BELA group, patients were receiving intravenous infusion of Belatacept at a 5mg/kg dose at 4-weekly intervals, associated with 500mg bid MMF and 5 mg/day of oral steroids.

**Assessment of Influenza (H1N1-HA)-specific memory B cells**

H1N1-HA was incubated with the dye at a molecular ratio of 1:10 for 1 hour at room temperature and then loaded into a Zeba Micro Spin Desalting column 7K (Thermofischer Scientific) to remove unbound dye. The degree of labeling was determined following the manufacturer’s instructions by measuring the absorbance of conjugated protein at the relevant wavelength by spectrophotometry.

# *Supplementary Figures and Tables*

**Supplementary figure 1. Representative gating strategy of H1N1 HA specific memory B cells and FACS plot examples.**

**1A:** Representative gating of B cell populations is shown. Briefly, mononuclear cells were gated out of all events followed by subsequent singlet gating. Live cells are gated using Live/Dead viability kit. B cells were then gated as CD19+, then B cells were further subdivided into naive B cells (CD27-IgD+), non–class-switched memory B cells (CD27+IgD+), double-negative B cells (CD27-IgD-) and Switched memory B cells (CD27+IgD-). HA positive cells (as well as human albumin as negative control) were gated in the memory B cell subset (CD19+, CD27+). Plasmablasts were gated as CD27+CD38^hi^ cells into CD19+.


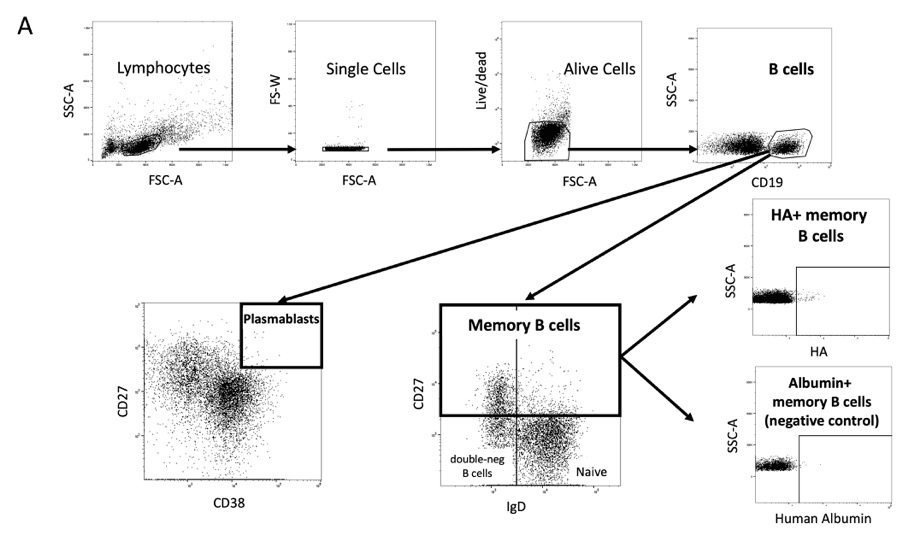
**
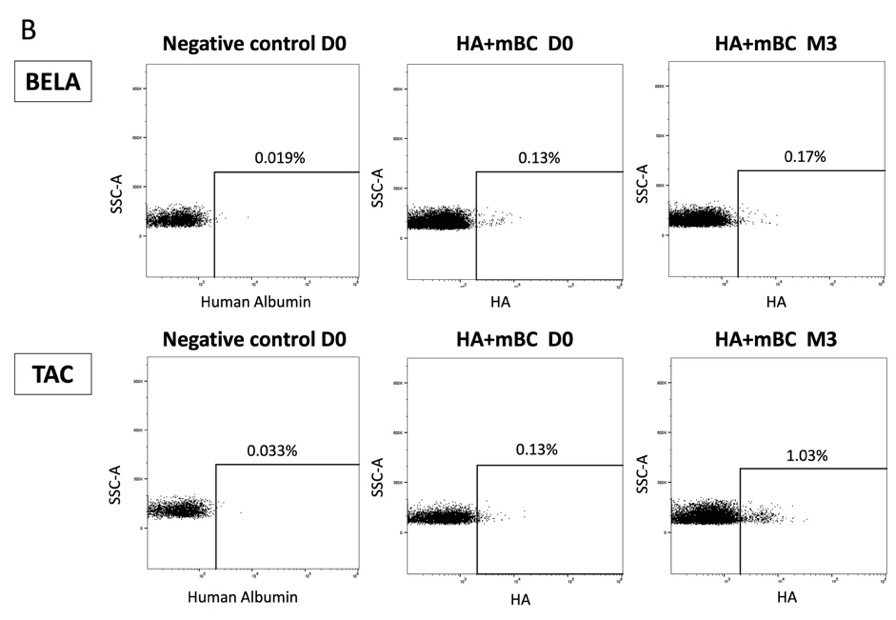
1B:** FACS plot examples gated in CD19+CD27+ memory B cells for negative controls (human albumin), HA at day 0 and at 3 months for one BELA patient and for one TAC patient

**Supplementary figure 2. Representative gating strategy of T follicular helper cells (Tfh) and FACS plot examples.**

**2A:** Representative gating of T follicular helper cells (Tfh) is shown. Mononuclear cells were gated out of all events followed by singlet gating. Live/Dead viability kit was used to gate live. Memory T cells were gated as CD4+ CD45RA-. Into memory T cells, Tfh were gated as CD45- CXCR5+. Into this subset, activated Tfh were gated as ICOS+ PD1+.

**2B:** FACS plot examples gated in memory T cells (CD4+ CD45RA) of Tfh (CD4+CD45RA- CXCR5+) and activated Tfh (CD4+CD45RA- CXCR5+ ICOS+ PD1+) gated on CD4+CD45RA- CXCR5+ Tfh at day 0 and day 10 for one BELA patient and for one TAC patient.

**
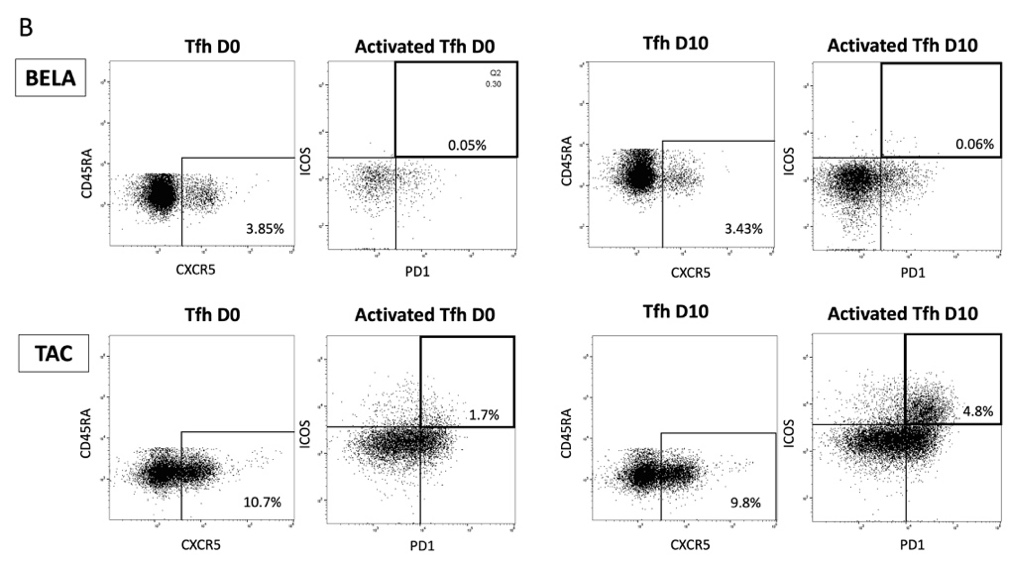

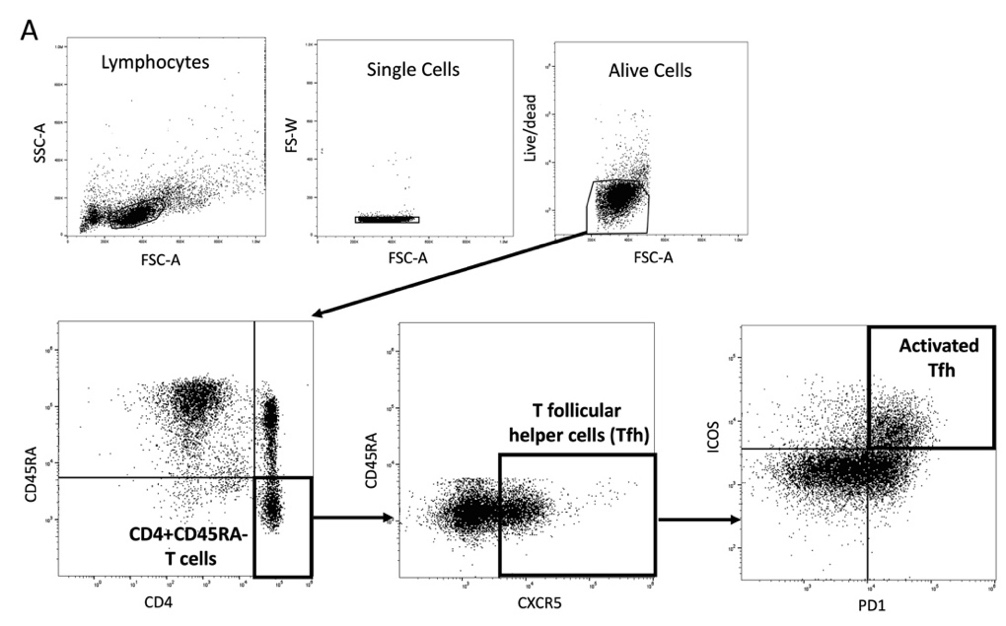
**

**Supplementary figure 3. Kinetics of different circulating B-cell subsets between groups prior and after vaccination**

**3A:** Kinetics of total CD19+ B cells;

**3B:** CD19+CD27+ mBC

**3C:**CD19+CD27+IgD- switched mBC

**3D:** CD19+CD27+IgD+ unswitched mBC

**
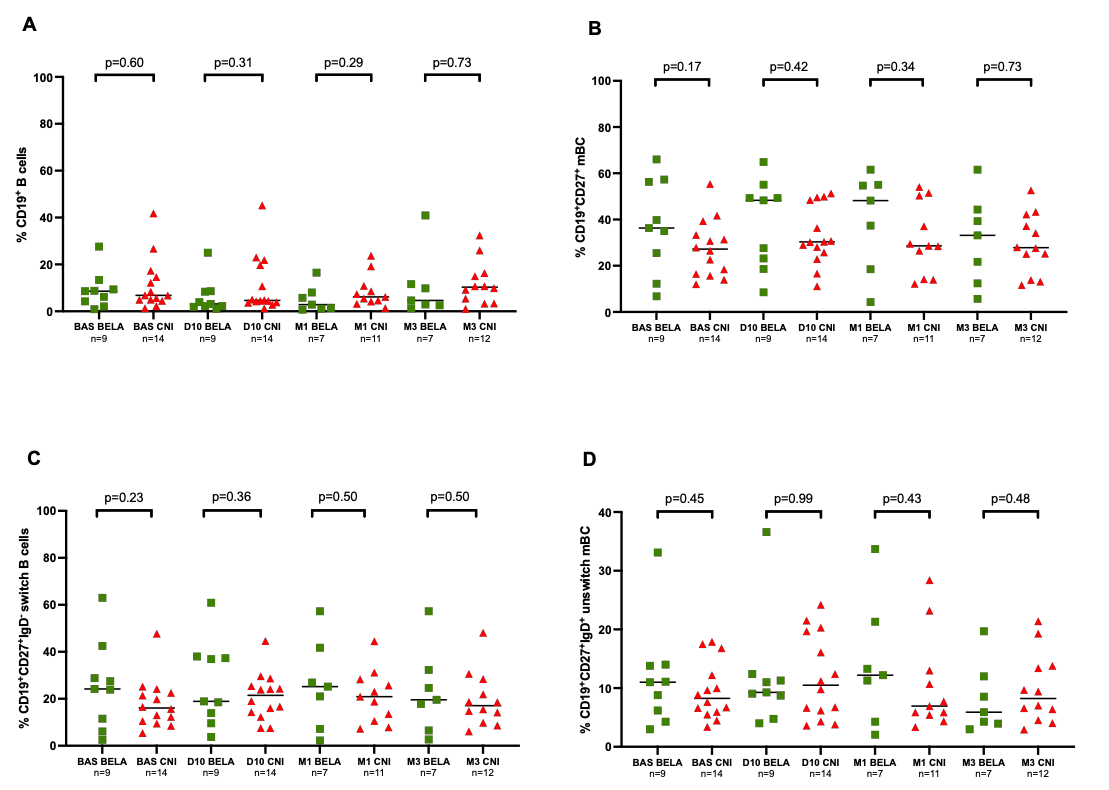
**

*The proportions of CD19^+^ B cells, CD19+CD27+ mBC, CD19+CD27+IgD- switched mBC and CD19+CD27+IgD+ unswitched mBC are expressed as a percentage in total lymphocytes.*

**Supplementary Figure 4:** Correlation between CD19^+^CD27^+^CD38^hi^ plasmablasts and CD4^+^CD45RA^-^CXCR5^+^ICOS^+^PD1^+^activated Tfh expansion between baseline and D10 between the two groups.

**
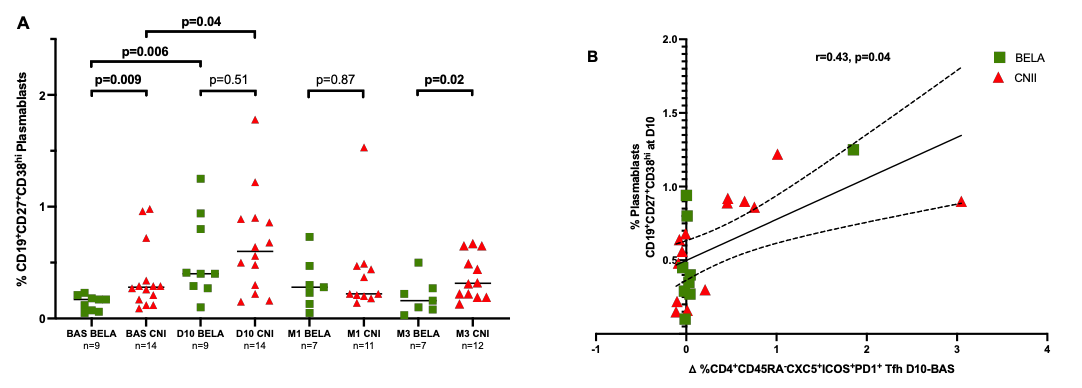
**

*The proportions of CD19^+^CD27^+^CD38^hi^ plasmablasts and CD4^+^CD45RA^-^CXCR5^+^ICOS^+^PD1^+^activated Tfh are expressed as a percentage in total lymphocytes.*
